# Side differences in NIRS-derived muscle oxygen saturation during treadmill running

**DOI:** 10.1007/s00421-026-06469-2

**Published:** 2026-10-14

**Authors:** Lara Zwiener, Oliver Richrath, Philip Skotzke, Benjamin Volkamer-Kien, Sascha Schwindling, Tim Meyer

**Affiliations:** 1 Institute of Sport and Preventive Medicine, Saarland University, Campus B8.2, 66123 Saarbrücken, Germany; 2 Olympic Training Center Rhineland-Palatinate/Saarland, Hermann-Neuberger-Straße 2, 66123 Saarbrücken, Germany

**Keywords:** Muscle oxygen saturation (SmO₂), Near-infrared spectroscopy (NIRS), Running, Side differences

## Abstract

**Purpose:**

Near-infrared spectroscopy (NIRS) offers a promising non-invasive method to assess muscle oxygenation in real time. While asymmetries in muscle oxygen saturation (SmO_2_) between dominant and non-dominant legs have been studied in cycling, data in running are limited. Such differences may affect NIRS use in training control. This study investigated SmO_2_ differences between dominant and non-dominant vastus lateralis during treadmill running at three workloads using a wearable continuous-wave NIRS device.

**Methods:**

Forty-eight participants (20 female, Age = 21.6 ± 1.5 years) performed two graded treadmill exercise tests, 14 days apart. The Moxy Monitor was placed bilaterally on the vastus lateralis to measure SmO_2_ and analysed across three individualized workloads (LOW, MID, HIGH). Linear mixed models were used with participant as random, and side and workload as fixed effects. To assess adipose tissue thickness (ATT) effects, a 7mm cut-off defined two subgroups (ATT<7mm, ATT>7mm). Bland-Altman plots examined mean bias and limits of agreement.

**Results:**

No significant side effects were found for either group (ALL p = .759, ATT<7mm p = .713 and ATT>7mm p = .890). Bland-Altman analysis revealed a small mean bias (ALL: -0.34%, ATT<7mm: − 0.48%, ATT>7mm: -0.22%) with wide limits of agreement between the legs.

**Conclusion:**

No systematic differences between SmO_2_ response between the legs were found. However, wide limits of agreement suggest that individual side differences may exist. A single NIRS device may sufficiently reflect bilateral SmO_2_ responses at the group level, while individual side-to-side-comparisons should be interpreted with caution.

## Introduction

Endurance sports require a balanced relationship between training and recovery to optimise adaptations and minimise injury risk (Etxebarria et al. 2019). Monitoring the internal load induced by training is therefore essential to adapt the training to the individual athlete. Bourdon et al. (2017)emphasized in their consensus statement the importance of monitoring exertion and individual load and outlined the most commonly used internal and external methods.

One method not included in their overview, but increasingly recognised in both research and practice, is near‑infrared spectroscopy (NIRS) (Perrey 2022). NIRS provides a non‑invasive, real‑time assessment of muscle oxygenation, distinguishing it from traditional approaches such as spiroergometry or blood lactate analysis. By emitting light in the near‑infrared spectrum (700–900 nm), NIRS quantifies the oxygenation of light‑absorbing chromophores in biological tissue (Barstow 2019). The Moxy Monitor (Fortiori Designs LLC, Hutchinson, MN, USA), a portable NIRS device designed for sports applications, estimates local oxygen saturation and total hemoglobin (Hb) by detecting scattered light signals. Its SmO₂ algorithm has been scientifically evaluated against arterial and venous occlusion-derived physiological calibration points, supporting the validity and reliability of SmO₂ values expressed on a 0–100% scale (Feldmann et al. 2019).

This technology enables continuous monitoring of tissue oxygenation during exercise.

Accurate application of the NIRS device requires knowledge of its validity, reliability, and potential lateral differences between extremities. Such information is particularly relevant when only one device is available and placement on a specific leg must be decided. Previous studies have examined differences between the dominant (DOM) and non‑dominant leg (NDOM) (Anliker et al. 2013; Girard et al. 2019; Hashizume et al. 2019; Iannetta et al. 2019; Nakahira et al. 2022; Skotzke et al. 2024). However, most focused on biomechanical or performance parameters. For instance, Iannetta et al. (2019) reported higher VO₂ values in DOM compared to NDOM during single‑leg cycling, with amplitudes of the deoxy‑hemoglobin (HHb) signal and SmO₂ correlating with mode‑specific VO₂. Similarly, (Carpes et al. 2010) observed pedaling asymmetries of 5–10% between legs at moderate workloads, although these differences were not consistently linked to leg dominance (Reinpõld & Rannama 2023).

While several studies have investigated SmO₂ asymmetries in cycling (Iannetta et al. 2019; Sendra-Pérez et al. 2025; Skotzke et al. 2024), evidence in running remains scarce. A recent systematic review (D’Hondt et al. 2024) analysing eight studies on inter‑limb asymmetry in running reported predominantly non‑significant or negative associations with performance, highlighting the controversial nature of this topic. Unlike cycling, where inter‑leg differences are typically small (<5%), running often exhibits asymmetries of 4–8% in variables such as ground contact time and force, reflecting its impact‑heavy and complex movement pattern.

Therefore, the primary objective of the present study was to examine differences in SmO₂ between the DOM and NDOM during treadmill running, using portable NIRS devices attached to the VL. Analyses were conducted across three exercise intensity domains (LOW, MID, HIGH) to assess bilateral SmO₂ responses across different physiological states. The central hypothesis was that SmO₂ would differ significantly between legs, with an expected medium effect size.

## Materials and methods

### Participants

Forty-eight sport science students participated in the study (see Table 1 for anthropometrics). Exclusion criteria were cardiopulmonary disease, acute infections, or injuries impairing treadmill running or coordination. All participants provided written informed consent. The study was approved by the Ethics Committee of the Faculty for Human Sciences, Saarland University (No. 24-13) and conducted in accordance with the Declaration of Helsinki. An a priori power analysis was conducted using G*Power (version 3.1, Heinrich Heine University Düsseldorf, Germany) based on an originally planned repeated-measures ANOVA. Assuming a large effect size (partial η^2^ = 0.15), an alpha level of 0.05, and a statistical power of 0.80, the calculation indicated a minimum required sample size of 46 participants. The present study was conducted as part of a larger research project, from which all complete and valid datasets meeting the predefined inclusion criteria were included. This resulted in a final sample of 48 participants, thereby exceeding the calculated minimum sample size and yielding a sample comparable to or larger than those used in previous studies investigating side differences in muscle oxygenation.

**Table 1:** Anthropometric data (All, ATT>7.5mm, ATT<7.5mm)

Anthropometric dates	All	ATT<7 MM	ATT>7 MM
Sample size	48	22	26
Sex (male/female)	28/20	20/2	8/18
Age (years)	21.6 (±1.5)	21.5 (±1.6)	21.8 (±1.4)
Height (CM)	174.0 (±8.1)	177.9 (±7.1)	170.7 (±7.4)
Weight (KG)	70.1 (±9.2)	71.4 (±7.1)	69.1 (±10.6)
Body Fat percentage (%)	17.2 (±4.1)	15.2 (±3.6)	18.9 (±3.8)
Left Leg dominance	5	3	2
Right Leg dominance	43	19	24
ATT DOM (MM)	7.8 (±3.1)	4.9 (±1.5)	10.2 (±2.0)
ATT NDOM (MM)	7.9 (±3.0)	5.2 (±1.3)	10.2 (±1.9)

### 2.2 Design and procedures

#### Experimental design

Participants performed two graded exercise tests (GXTs) on a treadmill (h/p cosmos pulsar, USA), scheduled 14 days apart (range 7–18 days) at the same time of day. During both trials, SmO₂ was measured bilaterally with one Moxy Monitor placed on DOM and one placed on NDOM. To avoid measurement interference, participants with unshaved body hair were instructed to shave their thighs (Barstow 2019).

#### Pre-exercise protocol

Dominant leg (“lateral preference”) (Carpes et al. 2010) was determined using the ball kick test (van Melick et al. 2017). Anthropometric data (weight, height, age) and body fat percentage were recorded. Skinfold thickness at the vastus lateralis (VL) was measured with a Harpenden caliper (Cintra-Andrade et al. 2023). The Moxy Monitor was then attached bilaterally at 2/3 of the distance between the anterior superior iliac spine and the patella, following SENIAM recommendations (SENIAM, n.d. 2026), and covered with a light shield.

#### Exercise protocol

GXTs were performed with a 1% incline, starting at 6 km/h with an increment of 2 km/h every three minutes. Each stage lasted 3 minutes to allow SmO₂ to reach a quasi-steady state (Yogev et al. 2023). Between stages, 45 seconds of passive recovery were provided for capillary blood lactate sampling from the earlobe. Workload increments were predefined and were not adjusted based on NIRS-derived signals. Perceived exertion was assessed at each stage using the Borg 6–20 scale (Borg 1970). Tests ended upon voluntary exhaustion.

### Measures

#### Adipose tissue thickness

Body fat percentage was determined from ten skinfolds (Parizkova 1961). Adipose tissue thickness (ATT) at DOM and NDOM VL was calculated as skinfold thickness (SF) × 0.5 (Barstow 2019).

#### Muscle oxygenation

SmO₂ was measured bilaterally using two wireless NIRS devices (Moxy Monitor, Fortiori Designs LLC, USA), a portable NIRS system designed for sports applications. The Moxy Monitor estimates local oxygen saturation and total hemoglobin (Hb) by detecting scattered light signals. Its SmO₂ algorithm has been scientifically evaluated against arterial and venous occlusion-derived physiological calibration points, supporting the validity and reliability of SmO₂ values expressed on a 0–100% scale (Feldmann et al. 2019). The device has also been validated and shown reliable across exercise tasks (Crum et al. 2017; Skotzke et al. 2024; Yogev et al. 2023).

NIRS uses near-infrared light (700–900 nm) and the modified Beer–Lambert law (Delpy and Cope 1997) to quantify oxygenation of haemoglobin (Hb) and myoglobin (Mb), the primary chromophores (Balaban et al. 1996; Barstow 2019). The Moxy Monitor emits light via four diodes and detects scattered signals with two detectors (12.5 and 25 mm), enabling calculation of total [Hb + Mb] and SmO₂.

Studies have consistently shown good to excellent test–retest reliability of the Moxy Monitor in cycling, with ICC values ranging from 0.77 to 0.99 across workloads and legs (Crum et al. 2017; Skotzke et al. 2024; Yogev et al. 2023), though all findings stem from cycling protocols.

### Data processing

NIRS data were recorded with the VO₂ Master Manager app (v0.41.1, VO₂ Master, Canada). For each stage and each side, SmO₂ was calculated as the average value after excluding the first 60 s and the final 10 s of the stage (Batterson et al., 2023). Skinfold values were averaged across both tests. Side comparisons of SmO₂ were analysed across three individualized workloads categories based on the lactate response during the GXT. Lactate thresholds were determined according to the approach described by Dickhuth et al. (1999). Briefly, LT1 was defined as the minimum in the lactate/speed ratio and LT2 as an increase in blood lactate concentration by 1.5 mmol/L. LOW was defined as the fully completed stage below the first lactate threshold (LT1), HIGH as the stage above the second lactate threshold (LT2), and MID as the stage located between LT1 and LT2. If an even number of stages was located between LT1 and LT2, the higher intermediate stage was selected. Thus, LOW, MID, and HIGH represented individualized physiological intensity domains, allowing the comparison across participants in a similar physiological state.

All values were stored for the total sample (ALL). Based on the methodological considerations described by McManus et al. (2018), participants were additionally stratified according to adipose tissue thickness using a 7mm threshold. Accordingly, subgroup analyses were performed for participants with ATT<7mm and ATT>7mm.

### Statistical analysis

Analyses were performed with jamovi (version 2.7.34, The jamovi project, Australia). Paired t-tests were used to assess ATT differences between DOM and NDOM. SmO₂ differences were examined using linear mixed models calculated separately for ALL, ATT>7mm, and ATT<7mm. SmO₂ was entered as the dependent variable, with Workload, Side, and the Workload × Side interaction as fixed effects and participant ID as a random intercept. To test our hypothesis, post-hoc comparisons for Side at each Workload were performed on the estimated marginal means. Bonferroni correction was applied.

Bland–Altman plots were used to assess agreement between DOM and NDOM by calculating mean bias and limits of agreement (Giavarina 2015). Missing SmO₂ values due to measurement error or NIRS probe detachment were not imputed; analyses were based on the available observations for the respective workload, side, and subgroup. For all tests, significance was set at p ≤ 0.05.

Of 576 expected SmO₂ values in the total sample (48 participants × 2 trials × 3 workloads × 2 sides), eight values were missing because the Moxy Monitor detached during treadmill running, most likely due to increased sweating and loss of skin adhesion. This corresponded to 1.39% of the total dataset, resulting in 568 SmO₂ values included in the final analysis. Four missing values occurred in ATT<7mm and four in ATT>7mm.

## Results

### Comparison of DOM and NDOM

The difference in ATT for all participants between DOM (M = 7.90 mm, SD = 3.01) and NDOM (M = 7.80, SD = 3.15) was 0.10 mm, 95% CI [-0.20, 0.40] and not significant: t (47) = 0.66, p_two-sided_ = 0.51. Similarly, for both subgroups no differences were found (p > 0.05).

### ALL

For the total sample, the linear mixed model showed no significant main effect of workload on SmO₂ (F(2, 515) = 1.87, p = .155), no significant main effect of side (F(1, 515) = 0.09, p = .759), and no significant workload × side interaction (F(2, 515) = 0.04, p = .962). Estimated marginal means were similar between DOM and NDOM across LOW, MID, and HIGH (DOM: 52.4%, 53.9%, and 54.3%; NDOM: 52.5%, 54.6%, and 54.4%, respectively). Bonferroni-corrected post-hoc comparisons showed no significant side differences within any workload category (all p_adj_ = 1.000), and workload comparisons within DOM and NDOM were also not significant after correction (all p_adj_ ≥ .567).

**Fig. 1:**
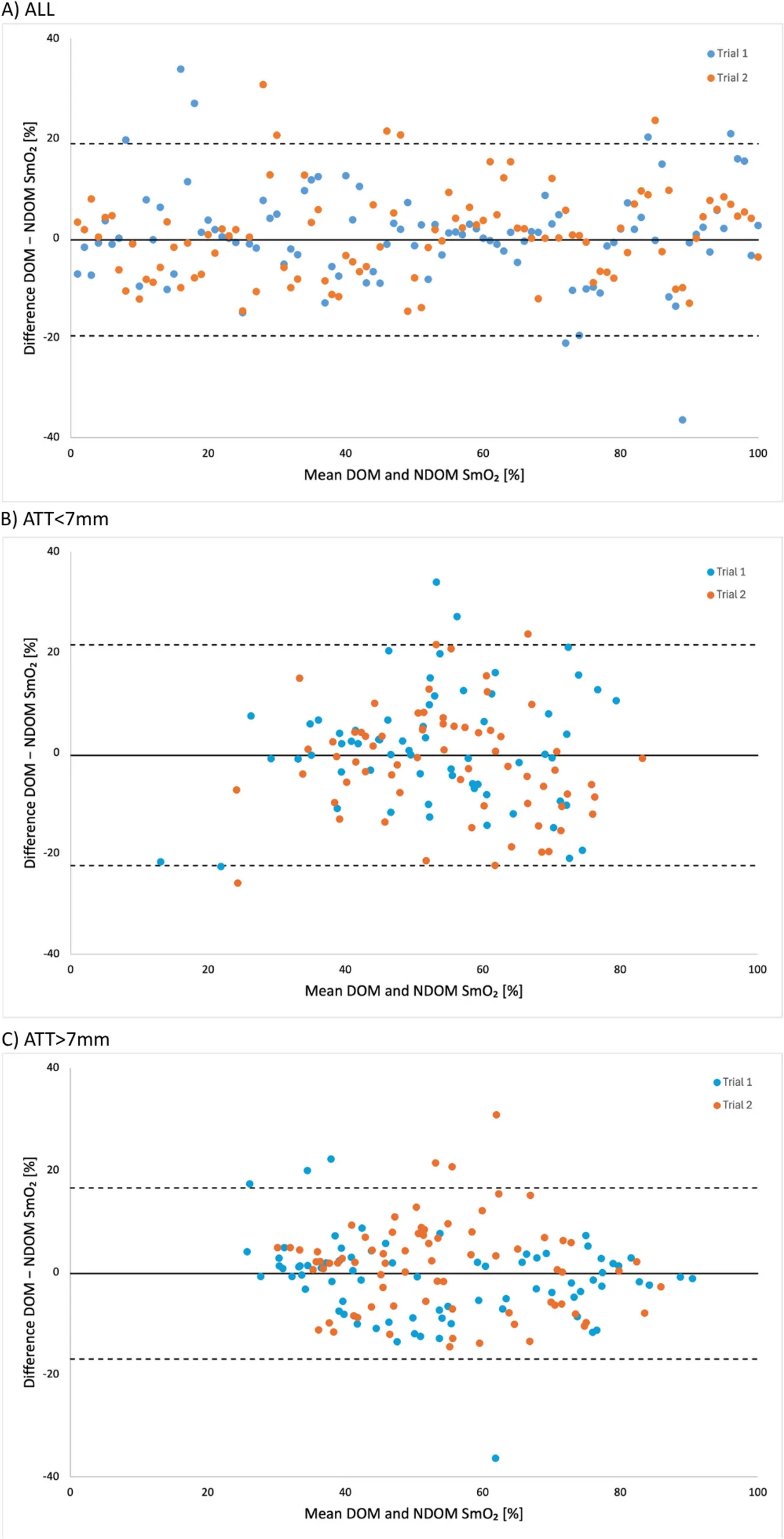
Bland–Altman plots illustrating the agreement DOM and NDOM SmO₂ values for **A** all participants, **B** ATT<7mm and **C** ATT>7mm. Differences were calculated as DOM − NDOM, withTrial 1 and Trial 2 displayed separately. Solid lines indicate the mean bias and dashed lines the upper and lower limits of agreement. Mean bias and LoA were −0.34% (−19.61 to 18.94%) for all participants, −0.48% (−22.45 to 21.45%) for ATT<7 mm, and −0.22% (−16.95 to 16.52%) for ATT>7 mm.

Figure 1 shows the Bland–Altman plot for the total sample. The mean bias was −0.34%, with lower and upper limits of agreement of −19.61% and 18.94%, respectively.

### ATT< 7mm

In the ATT<7mm subgroup, the linear mixed model revealed a significant main effect of workload on SmO₂ (F(2, 233) = 6.76, p = .001), but no significant main effect of side (F(1, 233) = 0.14, p = .713) or workload × side interaction (F(2, 233) = 0.01, p = .992). Estimated marginal means decreased from LOW/MID to HIGH in both legs (DOM: 55.7%, 54.7%, and 50.4%; NDOM: 56.1%, 55.4%, and 50.7%, respectively). Bonferroni-corrected post-hoc comparisons showed no significant side differences within any workload category (all p_adj_ = 1.000). Workload comparisons were not significant within DOM (all p_adj_ ≥ .054), whereas within NDOM, LOW differed significantly from HIGH (p_adj_ = .042).

Figure 1 shows the Bland–Altman plot for the ATT<7mm subgroup. The mean bias was −0.48%, with lower and upper limits of agreement of −22.45% and 21.49%, respectively.

### ATT > 7mm

In the ATT>7mm subgroup, the linear mixed model showed a significant main effect of workload on SmO₂ (F(2, 277) = 13.35, p < .001), but no significant main effect of side (F(1, 277) = 0.02, p = .890) or workload × side interaction (F(2, 277) = 0.03, p = .971). Estimated marginal means increased from LOW to HIGH in both legs (DOM: 49.5%, 53.3%, and 57.6%; NDOM: 49.4%, 54.0%, and 57.6%, respectively). Bonferroni-corrected post-hoc comparisons showed no significant side differences within any workload category (all p_adj_ = 1.000). Workload comparisons showed significant LOW–HIGH differences within both DOM and NDOM (both p_adj_ < .003), whereas LOW–MID and MID–HIGH comparisons were not significant after correction.

Figure 1 shows the Bland–Altman plot for the ATT>7mm subgroup. The mean bias was −0.22%, with lower and upper limits of agreement of −16.95% and 16.52%, respectively.

**Fig. 2:**
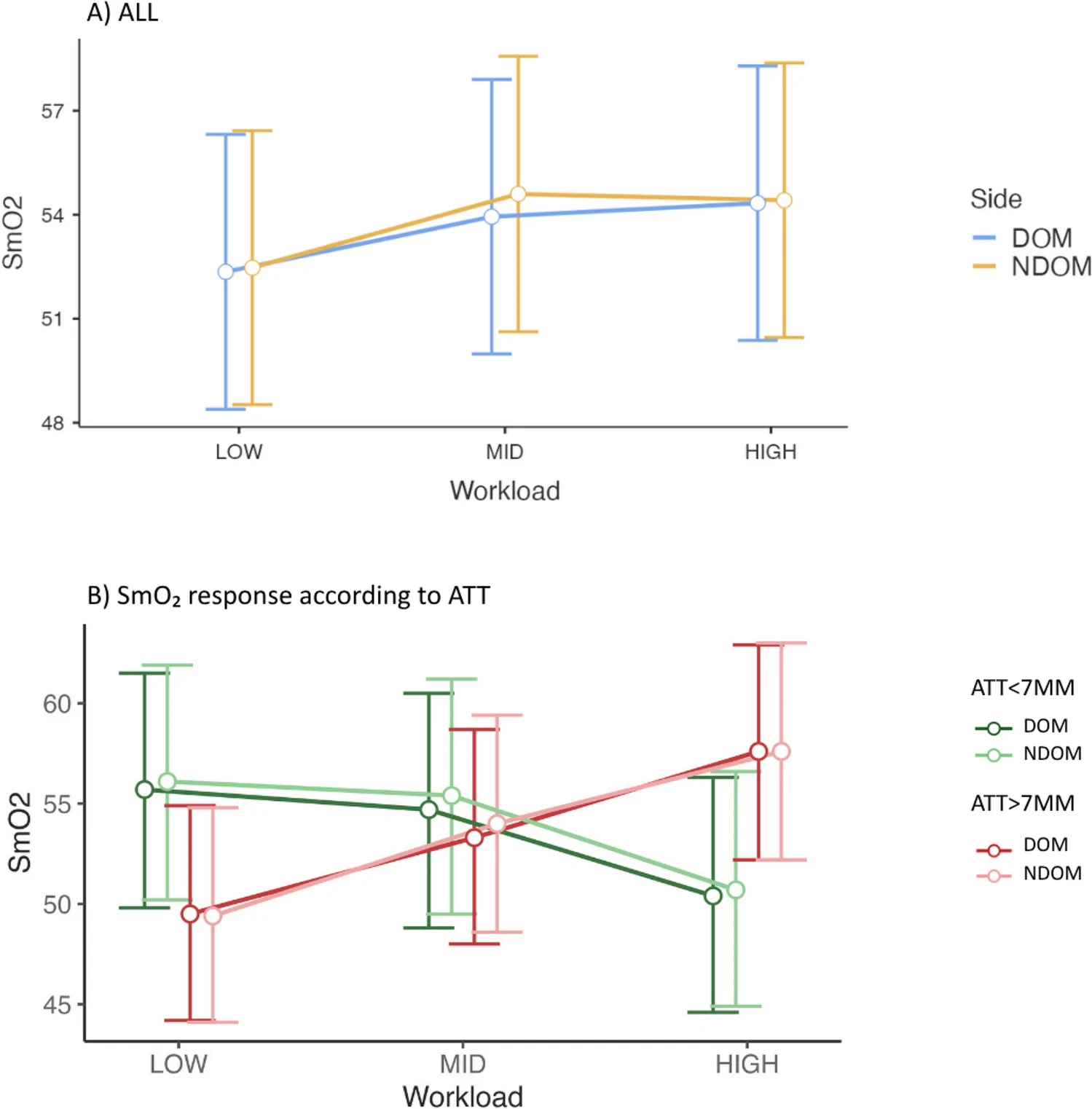
Estimated marginal means of SmO₂ across workload categories for the total sample (ALL; **A** and comparison between ATT thickness groups **B** Values are displayed separately for the dominant (DOM) and non-dominant (NDOM) leg. Error bars represent 95% confidence intervals derived from the linear mixed model.

The estimated marginal means derived from the linear mixed models are shown in Figure 2. In the total sample (Figure 2A), DOM and NDOM values were closely aligned across workload categories. When stratified by ATT (Figure 2B), the workload-related SmO₂ pattern differed between participants below and above the 7 mm cut-off.

## Discussion

### No systematic differences between DOM and NDOM

The aim of this study was to examine differences in SmO₂ during treadmill running between DOM and NDOM. We did not find systematic differences in SmO_2_ between the vastus lateralis of both legs: for neither ATT group the linear mixed model did not find significant side differences at any workload. This suggests that, at the group level, single-limb SmO_2_ measurements may provide a reasonable estimate of bilateral responses during treadmill running. Bland–Altman analysis revealed only small mean biases between DOM and NDOM across groups (ALL: −0.34%; ATT<7mm: -0.48%; ATT>7mm: -0.22%), indicating no systematic over‑ or underestimation of SmO₂. This was further supported by the linear mixed models, which showed no significant main effect of side and no workload × side interaction in the total sample or in either ATT subgroup.

### High interindividual variability in SmO_2_

The corresponding LoA were wide across all groups. Although 95% of data points fall within these limits, their distance from zero indicates considerable individual variability. Thus, while group‑level agreement appears acceptable, differences of approximately 17–22% between limbs may occur individually. Side‑to‑side comparisons should therefore be interpreted cautiously, especially in clinical or monitoring contexts where small changes are meaningful. The wide LoA between DOM and NDOM are unlikely explained by leg dominance alone. Skotzke et al. (2024) similarly reported broad LoA (± 19.9%) without systematic side differences during cycling. Variability may stem from sensor placement deviations, local adipose tissue thickness, skin and subcutaneous tissue contributions, and the relatively low sampling frequency of wearable NIRS devices (Barstow 2019; McManus et al. 2018; van Beekvelt et al. 2001). Hence, although consistent bilateral differences were absent, notable inter‑limb variability cannot be excluded in given individuals. When asymmetries occur, the choice of measurement leg becomes critical for accurate SmO₂ assessment, particularly in repeated monitoring.

### Comparison to other studies and exercise modes

No study has explored SmO₂ differences between DOM and NDOM during treadmill running specifically. Our findings are in line with the results from Sendra-Pérez et al. 2025and Skotzke et al. (2024) in cycling, suggesting no physiological side difference exist. They also support assumptions in studies measuring only one limb and extrapolating to the other (van der Zwaard et al. 2016; Vasquez Bonilla et al. 2023). However, contradictory evidence is provided: Reinpõld and Rannama (2023) reported significant side differences in desaturation kinetics between left and right VL in trained cyclists, with limited agreement. Bilateral averages approximated pulmonary oxygen uptake kinetics more closely, suggesting potential benefits of dual‑side NIRS. Yet, due to biomechanical and physiological differences between cycling and treadmill running, these findings cannot be directly generalized.

### Impact of ATT on data quality

McManus et al. (2018) showed that ATT>7mm may affect SmO_2_ measured with the specific NIRS device used. In the present study, the workload-related SmO₂ pattern differed between participants below and above this cut-off (Figure 2B), suggesting that ATT influenced the measurement response. In the ATT<7mm subgroup, SmO₂ showed the expected tendency to decrease toward HIGH, with a significant LOW–HIGH difference only within NDOM after correction. In contrast, the ATT>7mm subgroup showed an increase in SmO₂ from LOW to HIGH, with significant LOW–HIGH differences within both DOM and NDOM. This pattern appears physiologically unexpected during an incremental running test, where increasing intensity would generally be expected to increase muscular oxygen extraction, consequently reducing local muscle oxygenation. One possible explanation is that, in participants with higher ATT, the NIRS signal may have been less sensitive to changes in muscle oxygenation and more strongly influenced by skin and subcutaneous tissue (Barstow 2019; Niemeijer et al. 2017). During incremental exercise, increases in skin blood flow and temperature may further affect the optical signal and contribute to altered SmO₂ values (Davis et al. 2006). Contrary to our ATT>7m results, Hasegawa et al. (2024) observed SmO₂ decreases with intensity using the Humon Hex (ICC ≥ 0.84). This aligns with McManus et al. (2018), who found Moxy deviates from other NIRS devices when ATT exceeds 7mm.

### Practical implications and future research

Although the present models did not show a workload × side interaction, the wide LoA indicate that individual DOM–NDOM variability may still occur. Previous cycling studies suggest that asymmetries may vary with exercise intensity or task demands. Iannetta et al. (2019) reported higher VO₂-peak and performance in DOM versus NDOM during cycling, suggesting better peripheral adaptation in DOM based on larger HHb amplitudes and MLSSp. Carpes et al. (2010) found pedal asymmetries at three lower training zones, but none at VO₂-peak > 90%. In contrast, Crum et al. (2017) reported declining Moxy reliability at higher intensities due to ischemia and movement artefacts, with lower between-day reliability and increased variability at higher workloads.

Because endurance athletes commonly perform a large proportion of their training at low intensities, often below the first lactate threshold (Stöggl and Sperlich 2015), SmO₂ monitoring in this range is of practical relevance. In applied settings, however, SmO₂ is often assessed using only one NIRS device placed on one limb. The present findings suggest that this approach may be acceptable as no systematic side differences were observed. This suggests that in healthy athletes without any known side-imbalances SmO_2_ may be obtained from either leg. However, the broad LoA indicate that caution is needed when interpreting individual side-to-side differences. Future studies should examine whether individual side differences or signal variability are more pronounced within specific individualized workload categories or metabolic intensity domains, and whether single-limb monitoring is sufficient in this context.

To our knowledge, this is the first study to investigate SmO₂ differences between DOM and NDOM during running, whereas previous studies have mainly addressed cycling (Sendra-Pérez et al. 2025; Skotzke et al. 2024). The findings further support the use of NIRS during running exercise. Future studies should examine whether sport-specific movement patterns, particularly unilateral versus bilateral demands, influence leg dominance, potential SmO₂ asymmetries, or injury-related outcomes (Vasquez Bonilla et al. 2023).

## Limitations

As with all studies, limitations exist. Although the sample comprised 48 participants, only 22 had an ATT below 7mm (McManus et al. 2018), the majority of whom were male. Larger samples increase statistical power (Hopkins 2000), and sex imbalance can impair transferability. Nevertheless, participants with larger skinfolds (mostly females) showed results comparable to the other half. Hopkins (2000) emphasized that sample heterogeneity influences reliability and generalizability. While heterogeneity can increase variability needed to detect effects, homogeneous samples may better represent specific populations. Our study was heterogeneous in anthropometric data (Table 1) and endurance background. A strength is the high proportion of female subjects, though only two were in the ATT<7mm group. Future studies should include more females with an ATT<7mm (McManus et al. 2018), though such samples may be difficult to obtain outside of highly trained athletes. Nikolaidis et al. (2020) reported higher skinfold thickness in women (13.0 ± 3.4 mm) than men (9.9 ± 2.9 mm) at the patella, near the VL. Given these sex differences and evidence of potential differences in muscle oxygenation (Espinosa-Ramírez et al. 2021), female inclusion is crucial.

In some cases, the Moxy detached during GXT, leading to data loss. Detachment at higher intensities was likely caused by increased sweating and insufficient adhesive material, as well as greater impact and ground reaction forces. Future studies should ensure secure placement to minimize data loss during treadmill testing.

Another strength and weakness is the lack of control for exercise and diet between tests. While this may reduce reliability, it reflects real‑life conditions.

Furthermore, the present findings are limited to SmO₂ measurements of the vastus lateralis and should not be directly generalized to other muscles or measurement sites. Koga et al. (2007) demonstrated spatial heterogeneity of SmO₂ within the quadriceps during cycling, with variability in deoxygenation timing (CV: 3–56% and 2–21%) and primary deoxygenation kinetics (CV: 6–48% and 7–47%). Therefore, future studies should consider additional muscles and measurement locations when examining side differences. Nevertheless, measuring SmO₂ at the vastus lateralis remains appropriate for running due to its locomotor role and established NIRS validity (Feldmann et al. 2019).

## Conclusion

This study shows no significant differences in SmO₂ between DOM and NDOM during treadmill running across individualized workload categories. No significant side effect or workload × side interaction was found, suggesting that leg dominance does not systematically influence SmO₂ responses at the group level. Large LoA for side differences could not be explained by leg dominance alone. It remains to be investigated if these side differences reflect true physiological differences or result from methodological variability and measurement error. Thus, although consistent side differences were absent, substantial inter‑individual variability was observed.

## Data Availability

The datasets generated during the study are available from the corresponding authors on reasonable request.

## Abbreviations

ATT: Adipose tissue thickness
ANOVA: Analysis of variance
DOM: Dominant leg
NDOM: Non-dominant leg
GXT: Graded exercise test
Hb: Hemoglobin
LoA: Limits of agreement
Mb: Myoglobin
NIRS: Near-infrared-spectroscopy
SmO_2_: Muscle oxygen saturation
SF: Skinfold thickness
VL: Vastus lateralis muscle
